# Effectiveness of an Ecological Momentary Intervention for Reducing Risky Alcohol Consumption Among Young Adults: Protocol for a Three-Arm Randomized Controlled Trial

**DOI:** 10.2196/14190

**Published:** 2020-03-31

**Authors:** Cassandra Wright, Paul M Dietze, Emmanuel Kuntsche, Michael Livingston, Paul A Agius, Robin Room, Michelle Raggatt, Margaret Hellard, Megan S C Lim

**Affiliations:** 1 Burnet Institute Melbourne Australia; 2 Centre for Alcohol Policy Research La Trobe University Melbourne Australia; 3 School of Public Health and Preventive Medicine Monash University Melbourne Australia; 4 Behavioural Science Institute Radboud University Nijmegen Netherlands; 5 Department of Clinical Neurosciences Karolinska Institutet Stockholm Sweden; 6 Judith Lumley Centre La Trobe University Melbourne Australia; 7 Centre for Social Research on Alcohol and Drugs Stockholm University Stockholm Sweden; 8 Melbourne School of Population and Global Health University of Melbourne Parkville Australia

**Keywords:** alcohol, brief intervention, young adult, alcohol drinking, prevention and control, mobile phone

## Abstract

**Background:**

Recent research has investigated the utility of mobile phone–delivered interventions for reducing risky single-occasion drinking, also known as binge drinking. In the past five years, focus has been placed on ecological momentary interventions (EMIs), which aim to deliver intervention content in correspondence to real-time assessments of behavior, also known as ecological momentary assessments (EMAs).

**Objective:**

This study aims to assess the effect of a fully automated, tailored, mobile phone–delivered EMI termed Mobile Intervention for Drinking in Young people (MIDY) on young people's risky single-occasion drinking behavior.

**Methods:**

We will use a three-armed randomized controlled trial design to determine the impact of MIDY on peak consumption of alcohol among young people. A list of mobile telephone numbers for random digit dialing will be generated, and researchers will telephone potential participants to screen for eligibility. Participants will be randomized into one of three intervention groups. For 6 weeks, EMI, EMA, and attention control groups will complete hourly EMA surveys on their mobile phones on Friday and Saturday nights. EMI participants will receive personalized feedback in the form of text messages corresponding to their EMA survey responses, which focus on alcohol consumption, spending, and mood. EMA participants will not receive feedback. A third group will also complete EMA and receive feedback text messages at the same time intervals, but these will be focused on sedentary behavior and technology use. All groups will also complete a short survey on Saturday and Sunday mornings, with the primary outcome measure taken on Sunday mornings. A more detailed survey will be sent on the final Sunday of the 6-week period, and then again 1 year after recruitment.

**Results:**

The primary outcome measure will be an observed change (ie, reduction) in the mean peak number of drinks consumed in a single night over the 6-week intervention period between the EMI and attention control groups as measured in the weekly EMA. We expect to see a greater reduction in mean peak drinking in the EMI group compared to that in the attention control group. As a secondary aim, we will assess whether mean peak drinking is reduced in the EMA group compared to the attention control group. We will use a random-effects mixed-modeling approach using maximum-likelihood estimation to provide estimates of differences in peak drinking across time periods between those receiving the intervention (EMI) and attention control participants. An intention-to-treat approach will be taken for the analysis. Individuals and study groups will be modeled as random and fixed factors, respectively.

**Conclusions:**

This study extends our previous work investigating the efficacy of a mobile EMI (MIDY) for reducing risky drinking among young adults in Australia, and will add to the expanding literature on the use of mobile interventions for reducing risky alcohol consumption.

**Trial Registration:**

Australian New Zealand Clinical Trials Registration (ANZCTR): ACTRN12617001509358p; http://www.anzctr.org.au/ACTRN12617001509358p.aspx

**International Registered Report Identifier (IRRID):**

DERR1-10.2196/14190

## Introduction

Recent research has investigated the utility of mobile phone–delivered interventions for reducing risky single-occasion drinking (also known as binge drinking). Smartphones are ubiquitous in the lives of young Australians. A 2017 study showed that 94% of Australians aged 19-25 years spent more than 1 hour on their smartphone each day, with 63% spending more than 3 hours. Over half of the respondents in this age group reported that they check their phone every 15 minutes (27%) or every 30 minutes (25%) [1]. Most mobile phone–delivered alcohol interventions have focused on students and other young adults [2-11] , due to the high rates of alcohol consumption and alcohol-related harm in this population, in conjunction with their high rate of smartphone ownership [12,13].

Among the mobile phone–delivered interventions for alcohol consumption that have been documented in the literature to date, many take a brief intervention approach and provide some form of tailored feedback in response to screening of drinking behavior and other variables. Suffoletto and colleagues conducted several studies in which they recruited young adults from hospital emergency departments and sent them tailored advice via short message service (SMS) text messaging based on reported single-occasion drinking behavior, intentions to drink over the coming weekend, and commitment to reduce drinking and weekly drinking [3-6]. Suffoletto et al [5] reported small reductions in the drinking days and number of drinks per day over a 12-week period in the intervention group compared with those of the control group.

In the past five years, focus has been placed on ecological momentary interventions (EMIs), which aim to deliver the intervention content in correspondence to real-time assessments of behavior, also known as ecological momentary assessments (EMAs) [14]. The difference between EMI-based studies and the aforementioned mobile phone–delivered alcohol intervention studies is the act of interrupting the drinking event in the moment it occurs. EMIs frequently feed off information provided in EMAs, allowing for the intervention to be specifically tailored to the individual’s circumstances at the time. The combination of these two factors allows for a highly salient interruption at a time most pertinent to the recipient [14,15]. In our previous work, young adults likened the experience of receiving EMI to having a sober friend or a sober version of themselves pull them out of a drunken haze and gently tweak their behavior to help them avoid going “too far” [16].

Riordan et al [8-10] have focused on EMI for university students, particularly during the orientation week period. In two separate studies, they tested the effect of sending EMAs and EMIs during orientation week, and throughout the university semester; these EMIs were developed specifically for the student population and were informed by formative research. Their results have been mixed, with one study showing an effect in females but not males [8] and a second study showing an effect in one college but not another [9]. Although Riordan et al [17] highlighted a key risk group for alcohol consumption and related harm, many other nonstudent young adults also drink to excess regularly. Therefore, there is a need to test EMIs that focus on more general populations of young adults.

The current study extends our previous work in developing and testing a mobile phone–delivered EMI for reducing risky drinking in the event. Our 2016 study [16] outlined the co-design process taken to develop Mobile Intervention for Drinking in Young people (MIDY), during which 42 young people (adults 18-25 years old) participated in workshops to inform the delivery platform, timing, and frequency of assessment and intervention, questionnaires, tailoring process, and intervention content. The subsequent design included SMS text message–prompted, mobile Web-based EMAs (ie, an SMS text message with a link to a survey that opens in the Web browser of the mobile phone), with text-based feedback sent after each EMA is completed. Our feedback was informed by participants’ preferences in addition to motivational interviewing and brief intervention theory [18]. Hourly EMAs were preferred by most of participants, between 6 pm and 2 am on intervention nights. Participants requested feedback messages that were short, practical, and nonjudgmental. The same young people then tested the intervention on one night and completed follow-up interviews. The design was reported to be feasible and acceptable by almost all participants, with a response rate of over 90% for EMAs.

Subsequently, in 2018, we conducted a trial on the implementation of this same intervention among a sample of young people recruited from an existing cohort of young adults with risky drinking behavior [19]. We conducted a three-armed randomized controlled trial (RCT) to compare the full MIDY intervention with assessment-only and no-contact control groups. Participants were asked to complete the intervention or assessments on 6 nights over a 12-week study period. Our analyses showed a small and nonsignificant increase between baseline and follow-up data with respect to the mean number of standard drinks consumed at the most recent heavy-drinking occasion in the EMI group (12.5 vs 12.7). The EMA and control groups each showed a nonsignificant decrease (EMA 13.8 vs 11.8; control: 12.3 vs 11.6). There were no significant differences in these changes between the groups and effect sizes were small. We also did not observe differences between groups in other measures of alcohol consumption. However, this study was challenged by a small sample size relating to difficulties in recruiting participants from an ongoing cohort study into the intervention study, which affected our ability to detect significant effects. We therefore recommended further research into the efficacy of the MIDY intervention to clarify any effect on alcohol consumption.

Based on the two previous studies [16,19], this study aims to assess the effect of a tailored, mobile phone–delivered EMI (MIDY) on young people's risky single-occasion drinking behavior.

## Methods

### Study Design

We will use a three-armed RCT design to determine the impact of MIDY on peak consumption of alcohol among young people. The protocol for this study was registered with Australian New Zealand Clinical Trials Registry in October 2017.

**Figure 1:**
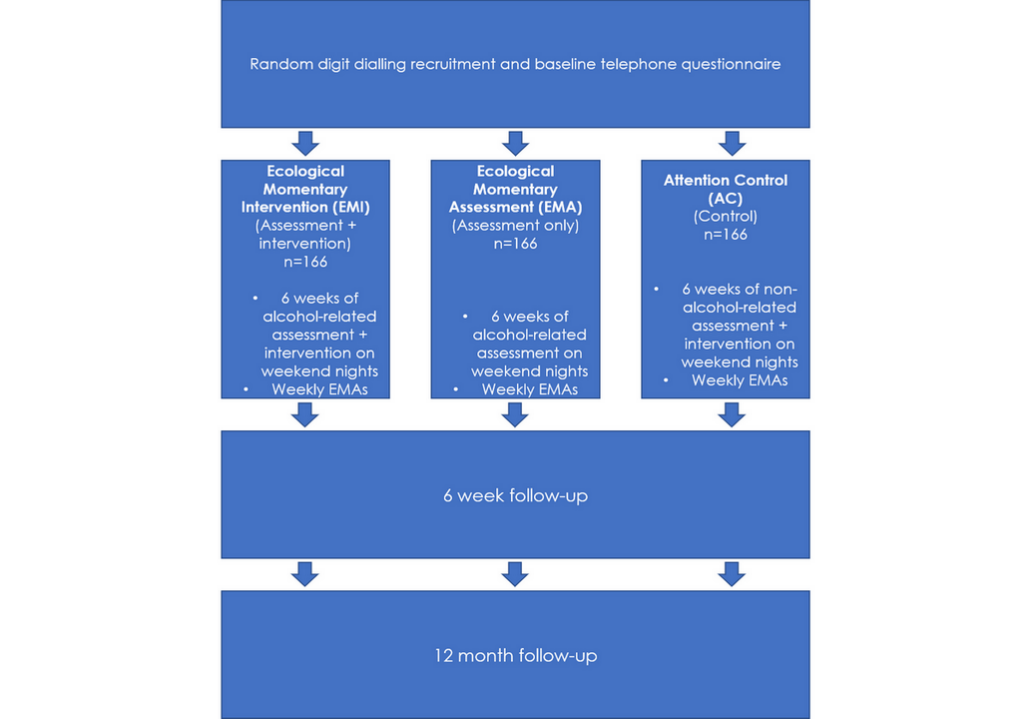
Study design.

The design of the study is outlined in Figure 1.

### Recruitment and Screening

Participants will be recruited using random digit dialing by trained researchers employed by TKW Research Group, a computer-assisted telephone interviewing (CATI) service provider. A list of mobile telephone numbers for random digit dialing will be generated by TKW Research Group, and their researchers will telephone potential participants to screen for eligibility.

Eligible participants will be Australian residents aged 18-25 years who report drinking at least 8 standard drinks in a single session at least once in the previous 12 months at screening, and possess a mobile phone with internet access. The alcohol consumption eligibility criteria were selected to capture a heavy drinking population of young people, and were based on the Young Adults Alcohol Study conducted by Dietze et al [20].

It is expected to take approximately 4 weeks to recruit the desired sample size of 500 eligible participants, with calls occurring on 5 days of each week in the late afternoon and evening, and a team of approximately 6-10 staff undertaking calls on each shift. TKW Research Group estimated that they will need to make approximately 10,000 calls to achieve this sample size, given that only a small segment of the population will meet the age criterion, and only half of those are expected to meet the drinking criterion. We have chosen CATI recruitment based on our previous success in using this method with this same age group [20], in which we recruited 800 young adults from Victoria, Australia for a cohort study with a similar length and intensity of recruitment fieldwork. We chose not to use SMS text messaging to directly recruit participants owing to (unpublished) discussions in formative stages of the research with young people who described that they would find being cold-contacted by researchers “dodgy” and that they would likely ignore the message.

When an individual is deemed eligible to be enrolled in the study, researchers will invite them to participate using a standard script to describe the study (ie, all three groups will receive the same information about completing mobile phone surveys on Friday and Saturday nights for the next 6 weeks). If they provide verbal consent, the researcher will administer the baseline questionnaire over the phone on the spot. Reasons for refusal will be documented. Following completion of the interview, TKW Research Group will forward the details of new participants to the Burnet Institute, and we will send a welcome SMS text message with a link to detailed study information relevant to the participants’ assigned arm; this will include a plain-language summary of the procedures relating to their trial arm, additional information as per the usual requirements of an “explanatory statement” required by an ethics committee, as well as some frequently asked questions. Participants will be blinded to the purpose of the study. The nature of the intervention means it will not be possible to blind participants to their allocated arm; however, they will remain unaware of the detailed procedures of the other arms.

### Event-Based Data Collection

#### Alcohol Ecological Momentary Assessment and Ecological Momentary Intervention Groups

Each participant from the EMA and EMI groups will fill out the same self-reported mobile phone–delivered surveys on Friday and Saturday nights for 6 weeks. In our previous studies [16,19], participants elected to complete the intervention on nights that they planned to drink. As the current trial includes an attention control (who are not exposed to alcohol questions or related content), this was not feasible, and we instead opted to predefine the intervention nights as the most popular drinking nights of young people based on our previous study. This was restricted to two nights to reduce the burden to the participants.

The design of the data collection procedures and intervention were informed by previous research [19,21,22] showing that this length and intensity of intervention were feasible. Our previous study with a 12-week intervention period retained 87/101 (85%) of the EMI participants at follow up [19]. We shortened this time period in the current study due to the above-mentioned decision to deliver the intervention on two nights of the week, rather than allowing participants to select their own intervention nights. We also found that hourly surveys were perceived as the optimal balance between minimizing the response burden and maximizing memory recall of alcohol consumption [16]. As with our previous study that tested an earlier version of this intervention [19], at 6 pm on study nights, participants will receive a short SMS text message asking them to complete a survey, containing a link to an online questionnaire. This 6 pm presurvey includes questions relating to their intentions for the night, such as their plans for the evening, who they are socializing with (if at all), their location, their mood, and if they plan to consume alcohol. If they plan to drink, they will be asked how much they plan to drink, spend, and eat; a ranked list of particular adverse events they wish to avoid (eg, vomiting, not being able to get home); their planned mode of transport home; next day plans; and any alcohol consumption so far. An addition to this version of the intervention is the ability for participants to set reminders that can be sent throughout the night by SMS text messaging; participants can select from a list of reminders (ie, “You have a study deadline!” or “Don’t forget that you have work tomorrow”) and can select the time for it to be sent. Multiple reminders can be set to come through between 7 pm that night and 11 am the next day.

Participants will then be sent shorter surveys at hourly intervals between 7 pm and 3 am, which ask about current location type, mood, spending, any alcohol consumption since the last survey, and, if consuming alcohol, their perceived drunkenness. Participants can stop the surveys for the night at any point by selecting an option at the end of a survey, or by replying to the number texting them with the word “stop.” Each time a survey is submitted, their GPS location is automatically collected.

At 11 am the next day (Saturdays and Sundays), participants will be sent another survey, which includes questions about any alcohol consumed or money spent after they went to bed, an estimated total standard drinks consumed and money spent for the night, an estimated volume of water consumed for the night, perceived social pressures to drink more and less, reporting of adverse events, “hangover” experienced (if they consumed alcohol), and a “fun” rating of the night.

#### Alcohol Ecological Momentary Intervention Group

The EMI, including questionnaires, message framing, and content, was developed in a participatory study with a group of 42 young adults in 2014 [22,23]. This three-part study involved half-day workshops to inform the design, individual testing of the intervention on a single night of drinking, and evaluation involving both in-depth interviews and a structured online survey. Further refinements were made following the implementation and evaluation of a pilot RCT [19,24] in 2015-2016. In conjunction with the co-design process, we have refined messages according to principles of motivational interviewing theory [18]. Each message incorporated an aspect of the FRAMES model, which includes Feedback (giving feedback on risks and negative consequences), Responsibility (emphasizing that the participant is responsible for making their own decisions), Advice (straightforward advice on modifying alcohol use); Menu of options (providing a menu of options to choose from), Empathy (demonstrating empathy and a nonjudgmental tone), and Self-efficacy (communicating optimism that the participant can modify their behavior if they so choose). We incorporated advice from participants in the formative stage to reduce the complexity of messages and move toward more straightforward feedback over the course of the night or as they consumed higher amounts of alcohol. The messages sent the following morning tended to focus more on encouraging the participants to reflect on their behavior and consider what they can choose to do differently the next time.

In line with EMI principles [14], the EMI group will additionally receive repeated interventions by SMS text messages each time they fill in a survey on intervention nights. These feedback SMS text messages comprise tips and advice for having a safe and enjoyable night, along with potential feedback related to cumulative drinking and spending. These messages are tailored to the individual based on their intentions, motivations, and plans reported in the presurvey and their current situation at the time of each hourly EMA during the night. Following completion of an EMA survey, a message is automatically selected from a bank of more than 2500 messages, each of which are “tagged” with particular labels that determine the individual and situations that they are appropriate for. For example, some messages are appropriate for particular locational contexts but not others, such as a message advising participants who are concerned about their spending to take out only a limited amount of cash from an automated teller machine, which is appropriate for venues or public settings but not private homes. Other tailoring variables include gender, their reported priorities for the evening, their transport plans, whether or not they have reported eating dinner, who they are with, how drunk they report feeling, and their mood. Raw data can also be directly dropped into the messages (ie, cumulative standard drinks reported so far) to provide more tailored feedback on drinking and spending.

We used a similar framework for tailoring our messages as adopted in our previous study [24] to determine the type or topic of message sent at each hourly interval, with decision logic based on different variables collected throughout the night. Our SMS text messaging system was developed by Questmetrics, which is linked with SurveyGizmo (the host of our EMA surveys) using webhooks, so that data from the surveys are immediately passed to the Questmetrics database and used to retrieve a relevant tailored message. For each survey filled out, algorithms are run within Questmetrics to match an individual’s responses against the logic framework that determines which message to send back to a participant. For example, at 1 am, if the participant has indicated that they plan to ride their bicycle home and that they feel drunk, they will receive a message suggesting that they make an alternative plan such as “It’s probably not safe for you to ride home tonight. What’s your backup plan?” A participant who reports in their presurvey that they would like to avoid having a hangover the next day and that they have not eaten dinner yet and plan to have more than 2 drinks that night may receive a message such as “Don’t want to spend tomorrow in bed? Dinner now is a great idea.”

EMI participants who respond to surveys reporting that they have not consumed any alcohol during the night will receive a generic response message such as “Thanks for your time so far!”

#### Alcohol Ecological Momentary Assessment-Only Control Group

The first control group (EMA) will follow the EMA data collection procedure described above (including registration for 6 weeks and event-based EMA for Friday and Saturday nights during the study period); however, they will *not* receive any feedback SMS text messages. This EMA-only group is required to examine potential reactivity to the EMA; that is, to assess the extent to which completing assessments alone (without any feedback or other intervention) can affect drinking behavior. Although previous studies have not found evidence of reactivity for EMA [25,26], in our pilot randomized control study, we noted a (nonsignificant) greater reduction in the primary outcome measure of peak drinks among participants in the EMA group compared to those in the EMI and no-contact control groups [19].

#### Attention Control Group

A second control group, attention control group, will fill out nonalcohol-related EMAs on weekend nights during the 6-week study period. We aimed to select a topic that is unlikely to have any influence on alcohol consumption, and instead focused on social interactions and sedentary behavior. Participants will be sent surveys on the same schedule previously described with weekly EMAs and event-based EMAs between 6 pm and 3 am on Friday and Saturday nights during the study period, with the option of opting out during the events. Participants will be asked to report on their plans for the evening, their social circle, social interactions throughout the night (including in person and online), and sedentary behavior such as how many minutes they have spent standing, sitting, or lying down in the past hour. They will then receive feedback SMS text messages in response to their surveys relating to their sedentary behavior, tailored by context such as social interactions and use of social media. There is a bank of several hundred messages available for this attention control group, similarly tagged with tailoring variables. For example, a participant who reported that their main plan for the night was watching TV or Netflix may receive a message such as: “Take a break from the TV every now and then to get up and stretch. You’ve been sitting down for 2 hours already tonight!” The attention control group will be the primary control group used for comparison to the EMI group in analyses.

### Reimbursement

All participants will receive reimbursements that are varied based on the level of participation in the study, with the baseline, 6-week follow up, and 12-month follow up incentivized in addition to the next-day surveys (11 am Saturday following a Friday night event, and 11 am Sunday following a Saturday night event). Each survey is worth AUS$5, with a bonus AUS$10 for completing the final Sunday survey at the end of the 6 weeks, and AUS$10 for the 12-month follow up. Total possible reimbursement is therefore AUS$80.

### Ethical Issues

Ethics approval for the RCT has been obtained from the Alfred Hospital Ethics Committee (project 18/18).

There is a small risk that participants will experience discomfort when answering questions about their alcohol consumption and its impacts, and reflecting on their previous weekend night. Participants do not have to answer any question if they feel uncomfortable about doing so.

There is also a small risk of participants feeling inconvenienced at having to answer hourly surveys on weekend nights. The impact is minimal given that the surveys will take only 1-2 minutes each. Participants are also be given the option at each hourly survey to opt out of completing subsequent assessments for that night.

Small payment will also be provided, as described above, to compensate participants for their time.

### Primary Outcome Measure

All measures were defined a priori. The primary outcome measure will be an observed change (ie, reduction) in the mean peak number of drinks consumed in a single night over the 6-week intervention period between those receiving the intervention (EMI) and attention control participants, as measured in the weekly EMA. This method improves on our previous study, which only collected this primary outcome measure at baseline and at follow up 12 weeks later.

The primary outcome measure is collected in the 11 am (next-day) survey on Sunday mornings each week during the 6-week study period (total of 6 measurements). The question asks participants to report on the highest number of alcoholic drinks consumed in a single occasion in the past week, and the night of the week alcohol was consumed. This measure will also be collected in a survey 12 months after commencement of the intervention period.

We expect to see a greater reduction in mean peak drinking in the EMI group compared to the attention control group. We also expect that the EMA group will show a reduction in mean peak drinking compared with that of the attention control group. The latter hypothesis is based on findings from our 2018 study, which showed a large but nonsignificant reduction in the EMA group compared to the control group [19]. We also expect to see a greater reduction in mean peak drinking in the EMI group compared to the EMA group.

It should be noted that this outcome is based on reducing peak consumption on a single occasion, rather than the general frequency or quantity of alcohol consumption over a period of time. This outcome is recognized as the most accurate measure of the key aim of binge-drinking interventions, namely to reduce the level of consumption (and harm) in an acute event. This single-item numerical measure has been shown to be reliable when compared to detailed time- and location-specific questioning across the drinking occasion [20].

### Secondary Outcome Measures

The following secondary outcomes of interest will be measured at the baseline, 6-week follow up (final Sunday 11 am survey), and 12-month follow up. Secondary alcohol consumption measures include annual consumption of >730 standard drinks per year (which equates to >2 standard drinks per day, in line with the Australian National Health and Medical Research Council guidelines for alcohol consumption) and, as two additional outcomes, monthly consumption of ≥5 and ≥11 drinks in a single session. These three measures will be derived from the graduated frequency measures, which include the following questions: “In the past 12 months, how often have you had 20 or more standard drinks in a day?” with response options including “Every day,” “5 to 6 days a week,” “3 to 4 days a week,” “1 to 2 days a week,” “1 day a week,” “2 to 3 days a month,” “About 1 day a month,” and “Less often than 1 day a month.” The question is then repeated with respect to the frequency of consumption: 11-19 standard drinks, 7-10 standard drinks, 5-6 standard drinks, 3-4 standard drinks, and 1-2 standard drinks. We will also examine changes in hazardous alcohol consumption using the Alcohol Use Disorders Identification Test [27].

We will measure experience of alcohol-related harms with yes/no/don’t know responses as to whether the participant has experienced different types of harm on their heaviest drinking occasion in the past 3 months, including: “Did you get into any verbal arguments or verbal fights on that occasion?”, “Did you fail to do what you intended to do the day after the session?”, and “Did you have any trouble getting home on that occasion?” These items were derived from the GenACIS [28] and VYADS questionnaires [29].

Usability and acceptability will be assessed in the follow-up survey among all groups using a 5-point Likert scale asking participants to rate a series of individual statements pertaining to their experience of undertaking the intervention (eg, “the assessments were easy to complete”). Additional process evaluation measures such as participant levels of response, refusal, and timeliness of response will also be explored to assess feasibility and acceptability.

### Randomization

We will use Stata statistical software package version 15 (StataCorp LLC, College Station, TX, USA) to conduct block randomization to ensure balanced randomization to each of the three study arms. Randomization will be undertaken by a researcher external to the study team.

### Effect Size and Sample Size

We hypothesize that the intervention will result in a decrease of 2.5 drinks on peak drinking

occasions. In our recent cohort study [17] that utilized similar inclusion criteria, the mean number of drinks consumed on the “most recent big night out” was 13.2 (SD 5.2). Reducing this by a mean of 2.5 standard drinks (Cohen *d*=0.48) would halve the estimated odds of alcohol-attributable mortality, motor vehicle accidents, and other serious injuries in the population [30,31]. Previous work suggested that this effect size is achievable: a nonrandomized mobile phone intervention found a mean reduction of 2.1 drinks per session following an untailored SMS text messaging–based intervention from a baseline of just 5.2 drinks [3]. This change is also consistent with a meta-analysis of alcohol brief interventions which found a significant aggregate effect size improvement in alcohol consumption of 0.67 (95% CI 0.40-0.95) 3 months after intervention and 0.26 (95% CI 0.20-0.32) 12 months after intervention [32].

Assuming a standard deviation of peak drinking of 5.2, conservative 10% end-point attrition, 90% power, and conservative 1% type-I error rate, we estimate that a sample of 145 participants per group is required at minimum to detect an effect size of this magnitude.

The study sample size is based on the primary aim, the associated clinically meaningful difference, and the proposed analytical methodology, which includes multiple comparisons of effect across the three groups of the trial. The sample size estimate has been calculated to test for a linear group-by-time interaction (ie, a greater mean reduction in number of drinks across the 6 weeks for those receiving the intervention) from a random-effects mixed-model analysis (6 measurements) and moderate correlations between subject measurements (*r*=.52, estimated from the variance parameters from our 2018 study [19]. In case of additional attrition, we will aim to recruit 500 participants.

### Statistical Analyses

We will use a random-effects mixed-modeling approach using maximum-likelihood estimation to provide estimates of differences in peak drinking across time between those receiving the EMI and attention control participants. An intention-to-treat approach will be taken for this analysis. Initially, individuals and study groups will be modeled as random and fixed factors, respectively, in these mixed-model analyses. Appropriate fixed terms for the functional form of the association between study time and peak drinking will also be estimated in modeling. Additional analyses will explore the model fit of estimation of study group and time factors as random effects. The interaction between group allocation and study time is our primary focus. These analyses will be repeated, as secondary analyses, to determine the impact of the assessments alone (ie, comparison of EMA and attention control groups) using participant observations from the EMA group. Simple main effects for both treatment and time will also be estimated in the models. Distributional assumptions of models will be tested in the data and appropriate transformations applied in cases where these are not reasonably met. All statistical analyses will be performed using Stata statistical software package version 15.

## Results

Recruitment for this study began in November 2018, and 601 participants were recruited by March 2019. We expect data collection for follow-up to be completed by March 2020.

## Discussion

### Overview

This study addresses unanswered questions from our previous research [19] about the efficacy of the MIDY intervention for reducing risky drinking behavior among young people. This research will add to the growing body of literature on EMI for alcohol use. If this intervention demonstrates efficacy in reducing alcohol consumption, we see potential for it to be offered to young people at crucial time points and events where risky drinking commonly occurs, such as during “schoolies” (a celebration following the completion of high school) or “Orientation week” (the first week of university for Australian and New Zealander students). We envisage that this intervention could either be made publicly available for young people to self-select into or be implemented in targeted populations.

### Limitations

This study also has a number of limitations. In our previous work, we encountered challenges with respect to recruitment, which participants described as relating to research fatigue after participating in several waves of a cohort study prior to being invited into the trial. However, it is also possible that the intervention itself is deemed unappealing or too burdensome to complete during social events, which could affect our ability to recruit an adequate number of participants.

Due to logistical considerations and funding restrictions, we opted to recruit and administer the baseline questionnaire via CATI, with the subsequent primary outcome assessments performed using mobile Web-based questionnaires. It is possible that changing the mode of administration may induce respondent bias. However, all three groups will be subject to the same biases.

As with most alcohol-related research, this study relies on self-reported data, which is prone to reporting bias; however, this bias is not expected to differ among the RCT arms. The primary outcome will be collected at a time when participants are unlikely to be under the influence of alcohol consumption.

It is difficult to blind participants in this type of intervention study; however, our inclusion of the EMA group should allow us to account for expectancy effects whereby participants may deduce that the purpose of the intervention is to monitor and intervene on drinking behavior.

As is the case for all intervention studies using incentives, adherence to the intervention may not be able to be replicated in real-world settings.

### Conclusion

This study extends our previous work investigating the efficacy of a mobile EMI, MIDY, for reducing risky drinking behavior among young adults in Australia, and will add to the proliferating literature on the use of mobile interventions for reducing risky alcohol consumption.

## Appendix

Multimedia Appendix 1 CONSORT-eHEALTH checklist (V 1.6.1).

## Abbreviations

CATI: computer-assisted telephone interviewing
EMA: ecological momentary assessment
EMI: ecological momentary intervention
FRAMES: Feedback, Responsibility, Advice, Empathy, Self-efficacy
MIDY: Mobile Intervention for Drinking in Young people
NHMRC: National Health and Medical Research Council
RCT: randomized controlled trial
SMS: short message service
